# Attentional Bias, Alcohol Craving, and Anxiety Implications of the Virtual Reality Cue-Exposure Therapy in Severe Alcohol Use Disorder: A Case Report

**DOI:** 10.3389/fpsyg.2021.543586

**Published:** 2021-02-22

**Authors:** Alexandra Ghiţă, Olga Hernández-Serrano, Jolanda Fernández-Ruiz, Manuel Moreno, Miquel Monras, Lluisa Ortega, Silvia Mondon, Lidia Teixidor, Antoni Gual, Mariano Gacto-Sanchez, Bruno Porras-García, Marta Ferrer-García, José Gutiérrez-Maldonado

**Affiliations:** ^1^Department of Clinical Psychology and Psychobiology, University of Barcelona, Barcelona, Spain; ^2^Department of Physical Therapy, EUSES - University of Girona, Girona, Spain; ^3^Department of Cognition, Development and Educational Psychology, University of Barcelona, Barcelona, Spain; ^4^Addictive Behaviors Unit, Hospital Clinic of Barcelona, Barcelona, Spain; ^5^Department of Physical Therapy, University of Murcia, Murcia, Spain

**Keywords:** alcohol use disorder, craving, anxiety, attentional bias, virtual reality cue-exposure therapy, eye-tracking

## Abstract

**Aims**: Attentional bias (AB), alcohol craving, and anxiety have important implications in the development and maintenance of alcohol use disorder (AUD). The current study aims to test the effectiveness of a Virtual Reality Cue-Exposure Therapy (VR-CET) to reduce levels of alcohol craving and anxiety and prompt changes in AB toward alcohol content.

**Method**: A 49-year-old male participated in this study, diagnosed with severe AUD, who also used tobacco and illicit substances on an occasional basis and who made several failed attempts to cease substance misuse. The protocol consisted of six VR-CET booster sessions and two assessment sessions (pre- and post-VR-CET) over the course of 5 weeks. The VR-CET program consisted of booster therapy sessions based on virtual reality (VR) exposure to preferred alcohol-related cues and contexts. The initial and final assessment sessions were focused on exploring AB, alcohol craving, and anxiety using paper-and-pencil instruments and the eye-tracking (ET) and VR technologies at different time points.

**Results**: Pre and post assessment sessions indicated falls on the scores of all instruments assessing alcohol craving, anxiety, and AB.

**Conclusions**: This case report, part of a larger project, demonstrates the effectiveness of the VR-CET booster sessions in AUD. In the post-treatment measurements, a variety of instruments showed a change in the AB pattern and an improvement in craving and anxiety responses. As a result of the systematic desensitization, virtual exposure gradually reduced the responses to significant alcohol-related cues and contexts. The implications for AB, anxiety and craving are discussed.

## Introduction

Abstinence maintenance remains one of the greatest challenges in alcohol use disorder (AUD) treatment. Previous findings demonstrated that the interplay between cognitive bias of processing alcohol content [e.g., attentional bias, (AB)], affective mechanisms and implicit elicitation of alcohol craving contribute to the development and maintenance of AUD (Manchery et al., 2017; Anker et al., 2018). As a result of excessive alcohol use, AB reflects an automatic processing response that prioritizes alcohol-related stimuli over other environmental stimuli (Field et al., 2014; Wilcockson et al., 2019) and triggers alcohol craving, described in the literature as an intense urge to consume alcohol (Drummond, 2001). In addition to AB, alcohol craving is directly triggered by affective mechanisms such as stress and anxiety (McCaul et al., 2017; Anker et al., 2018), particularly when alcohol cues are present (Sinha et al., 2011) and results in a repertoire of approach or drinking behavioral tendencies (van Lier et al., 2018).

There are several methods depicted in the literature that aim to explore alcohol cravings, such as neuro-psychophysiological instruments (Quintana et al., 2013; Tabakoff and Hoffman, 2013), self-reports (e.g., questionnaires; Kavanagh et al., 2013), cognitive and/or behavioral tasks (Franken, 2002; Field et al., 2008; Kavanagh et al., 2013; Schlauch et al., 2013; Lee et al., 2014; Kim and Lee, 2015), some of them in combination with the cue-exposure paradigm. The cue-exposure paradigm generally involves *in vivo*, imagery or multimedia exposure to alcohol-related cues and contexts to explore different underlying mechanisms of AUD, including alcohol cravings (Mellentin et al., 2017a). Such method emerged into the cue-exposure therapy (CET) approach, which aims to extinguish responses to alcohol craving by following the principles of systematic desensitization (Mellentin et al., 2017b). This therapeutic approach involves prolonged and repeated exposure to alcohol cues and the ultimate goal is to gradually reduce psychophysiological, affective, and cognitive responses to alcohol craving (Vollstädt-Klein et al., 2011). Although this psychological intervention has been employed in many studies, two meta-analyses concluded that CET in AUD show inconsistent results and limited positive outcomes (Conklin and Tiffany, 2002; Mellentin et al., 2017a). This is mainly because the therapy sessions involved exposure to one alcohol cue at a time (e.g., one bottle/glass of alcoholic beverage), within a clinical context, which delays generalization of therapy effects into naturalistic settings.

Emerging technologies like virtual reality (VR) and eye-tracking (ET) may complement existing methods in terms of assessment and treatment instruments applied in mental health (Segawa et al., 2020). These technologies provide high levels of ecological validity that approximate laboratory and naturalistic observations and its outcomes (Parsons, 2015; Segawa et al., 2020; Simon et al., 2020). For instance, VR can add effectiveness to conventional CET as a result of its fully enriched and immersive real-life simulations, which enhances the sense of presence within the VR environments (Hone-Blanchet et al., 2014; Ghiţă and Gutiérrez-Maldonado, 2018; Segawa et al., 2020). In AUD, VR has been implemented as an assessment instrument to elicit alcohol craving (Bordnick et al., 2008; Ryan et al., 2010; Kim and Lee, 2015) and as a treatment tool to reduce craving responses related to alcohol content (for example, see Hyun et al., 2013; Son et al., 2015). These studies using the VR technology have shown consistent results in terms of elicitation or reduction of subjective craving for alcohol (Ghiţă and Gutiérrez-Maldonado, 2018).

Regarding the use of the ET technology, it has been implemented mainly as an assessment instrument to explore patterns of eye-movement activity in relation to different AUD mechanisms like AB, drug craving, approach-avoidance tendencies (for instance, see Wilcockson et al., 2019). More recently, ET has been studied as a cognitive training instrument to modify AB toward motivationally salient stimuli (Mondino et al., 2020). Hence, ET parameters like first fixation, dwell time, or number of fixations derived from the individual’s oculomotor activity, are among the robust variables to detect AB toward alcohol content (Maurage et al., 2020). First fixation is the initial orientation or the very first gaze (fixation) point toward salient stimuli and depicts an implicit, automatic, early-stage attention processing (Schoenmakers et al., 2008). Number of fixations reflects the number of times an individual made a fixation toward one of the stimuli (Frings et al., 2018). Dwell time is understood as the total time observing a certain stimulus (Schoenmakers et al., 2008). Both number of fixations and dwell time variables reflect the maintenance stage of attention processing.

Considering the growing interest in digital health applications, the current study is part of a larger project entitled “*ALCO-VR: Virtual Reality-based protocol for the treatment of patients diagnosed with severe alcohol use disorder*,” which aims to test the effectiveness of Virtual Reality Cue-Exposure Therapy (VR-CET) in patients diagnosed with severe AUD. The first study in this project was focused on the identification of alcohol-related cues and contexts that trigger craving, in order to create clinically significant virtual environments (Ghiţă et al., 2019a). The results of that study indicated that the most frequent alcohol-related contexts were a bar, a restaurant, a pub, and at-home environments. The patients self-reported a wide range of alcoholic beverages and we included a menu of 22 alcoholic drinks in the VR platform. Hence, based on patients’ experiences, the second study of the project emphasized the development and validation of the “ALCO-VR” platform (Ghiţă et al., 2019b). A third study carried out as part of the project aimed to develop an AB assessment task using the ET technology. In this study, a visual attention task (VAT) was created to explore gaze patterns toward alcohol-related images vs. neutral images (Ghiţă et al., 2019c). The results of the third study, the VAT explored with the ET technology, were introduced in the last study of the project as a pre-post measurement of the efficacy effectiveness of VR-CET with the aim of exploring changes in gaze patterns. The final study in the project, a multi-site clinical trial, is currently implementing the outcomes of the previous studies to test the effectiveness of VR-CET in AUD patients (Hernández-Serrano et al., 2020). The main objective of the trial is to explore the clinical potential of VR-based therapy vs. treatment-as-usual (TAU) in AUD patients. At the moment of reporting this study, the clinical trial was an ongoing study.

The current study is a case report of a patient diagnosed with severe AUD, considered resistant to TAU, who took part in the last study of the ALCO-VR project, the clinical trial. The objective of the current study was to test the feasibility of the VR-CET protocol within the clinical trial. Once the patient completed the VR-CET program, our research group increased the sample size and are currently comparing VR-CET vs. TAU in AUD patients. Repeated, systematic and gradual exposure to VR alcohol cues and environments was performed as part of the psychological intervention (six VR-CET sessions). AB, alcohol craving, and anxiety levels were explored with different instruments before and after the VR-CET administration. Momentary levels of alcohol craving and anxiety were also examined during the six VR-CET sessions. Changes in AB patterns and reductions in alcohol craving and anxiety were expected at post-VR-CET assessment.

## Case Description

### Patient A

#### Clinical and Personal History

Patient A was a 49-year-old divorced man, father of two children, with an elementary educational background and low socio-economic status. He was the owner of two small bars. Family members with problematic use of substances included patient’s father (alcohol), uncle (alcohol), two brothers (alcohol, opioids, and stimulants), and previous partner (alcohol). The age of onset for alcohol consumption was 13. At the age of 16, patient used stimulants (cocaine) and cannabis for the first time. The age of onset for using opioids (heroin), hallucinogens (Lysergic acid diethylamide, LSD), and other stimulants (amphetamine) was 19. From the age of 20 until the age of 38, patient A underwent eight in/outpatient treatments for substance use disorders at a different hospital in Barcelona, Hospital Sant Pau. Treatment at this hospital included individual psychotherapy, group therapy, and pharmacotherapy (disulfiram, anxiolytics, and antipsychotic medication). Patient A maintained abstinence during treatments only, followed by alcohol abuse after treatment discharges.

#### Treatment at Hospital Clinic of Barcelona

At the age of 38, patient A received treatment for liver cirrhosis at Hospital Clinic of Barcelona, and he was derived then to the Addictive Behaviors Unit from the same hospital. Patient had a first appointment with a clinical psychologist but abandoned the treatment soon after. Between the age of 38 and 42, it is unknown whether the patient received treatment at a different hospital, either at public or private settings. At the age of 42, patient A had an overdose as a result of a combination of several substances: alcohol, stimulants, hallucinogens, and antipsychotic medication. After discharge from the intensive care unit for addressing the overdose episode, patient had a continuous pattern of alcohol use. At 46, patient A was administered two inpatient-based treatments for alcoholic hepatitis at Hospital Clinic of Barcelona. He had a 9-day abstinence period between these treatments for alcoholic hepatitis, followed by alcohol abuse after the second treatment discharge. Patient was again directed to the Addictive Behaviors Unit at the same hospital. At the second appointment with the clinical psychologist, patient A reported several motives to cease substance misuse: a hazardous pattern of substance use, severe alcohol withdrawal syndrome, numerous failed attempts to maintain abstinence over a prolonged period of time, severe alcohol craving, alcohol tolerance [patient A was consuming 26 standard drink units (SDU)/day at the time of the appointment at the age of 46], alongside using other illicit substances and tobacco on an occasional basis. A SDU is a single consumption of 10 g of ethanol (the standard quantity of wine or beer and half the standard quantity of liquor; Llopis Llácer et al., 2000). In addition to substance misuse, patient A reported psycho-social and economic concerns (divorce, family substance abuse, and unemployment).

#### Psychiatric Assessment

Patient A was assessed by different professionals at the Addictive Behaviors Unit (clinical psychologist and psychiatrist). Patient A fulfilled the criteria for antisocial personality disorder. In addition, the outcomes of the assessments indicated severe alcohol use disorder, moderate tobacco use disorder, moderate stimulant use disorder (cocaine), mild cannabis use disorder, mild sedative, hypnotic, or anxiolytic use disorder (as a result of continuous use of benzodiazepine-based medication) according to the Diagnostic and Statistical Manual of Mental Disorders (American Psychiatric Association, 2013). As patient A experienced numerous relapses after treatment discharges and even had a continuous alcohol consumption pattern during outpatient-based treatment, he was considered “resistant to TAU.”

#### Treatment-as-Usual at the Addictive Behaviors Unit

Patient underwent a detoxification program on an inpatient basis with clonazepam. Then pharmacotherapy included disulfiram, anxiolytic and gabapentin medication on an outpatient basis (disulfiram and anxiolytic medication from 46 to 49 years and gabapentin for several months at the age of 49). The psychological treatment followed a multidisciplinary protocol, including cognitive-behavioral, motivational, and third-wave therapeutic approaches at an individual and group level. Urine analyses were collected tri-weekly. From the age of 46 until 49, while under TAU at the Addictive Behaviors Unit, patient A was admitted to several therapeutic communities in Barcelona but abandoned after a maximum of 3–4 weeks. Despite the efforts from a multidisciplinary approach, patient A had a maximum of 4 weeks of abstinence each time there was an attempt to cease substance use, from 46 until the age of 49 as self-reported the patient and confirmed by urine analyses. He experienced several relapses mainly with alcohol, and occasionally with cocaine and cannabis. Overall, patient A had a continuous and hazardous pattern of alcohol consumption and occasional use of illicit substances from the age of 16 until 49.

#### VR-CET Program

**Figure 1:**
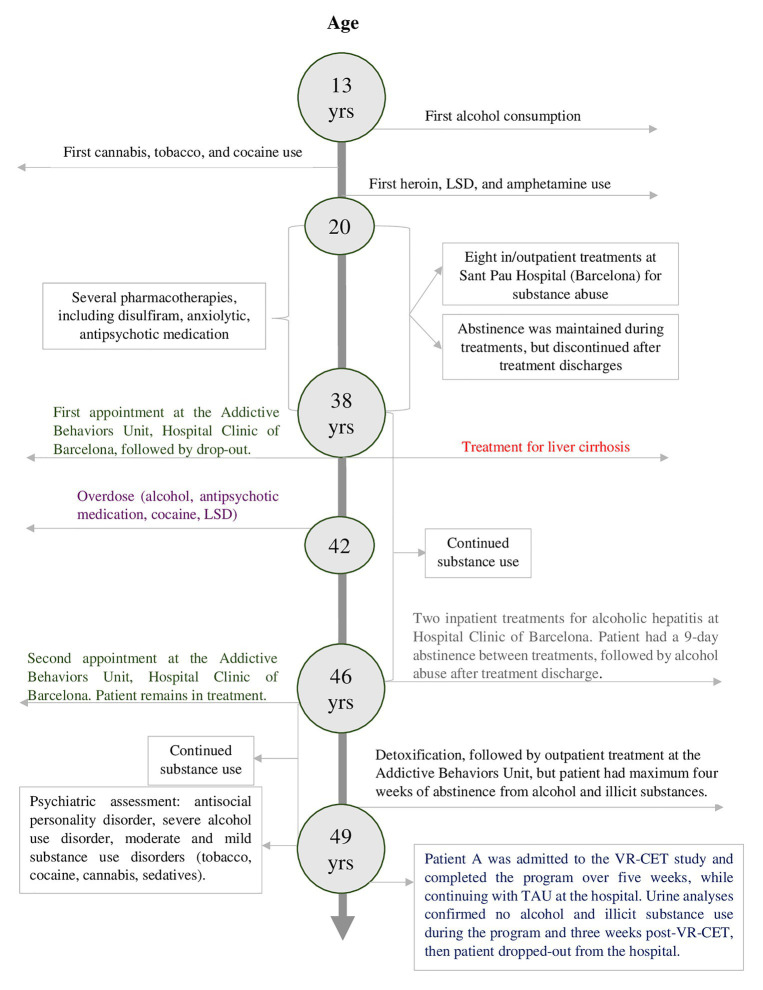
A timeline of patient’s relevant current and past clinical data.

At the age of 49, patient A was introduced to the VR-CET program as part of a larger clinical trial [the ethical code number of the clinical trial is 0377 (HCB/2017/0377) and the approval date was 09/2017]. Until that moment, patient A had never experienced a VR-based psychological treatment. At the moment of his inclusion in the study, patient A was under outpatient treatment at the Addictive Behaviors Unit, receiving medication and psychological treatment as detailed in the *TAU at the Addictive Behaviors Unit*. A month before the first appointment with the clinician-scientist from the University of Barcelona, patient A used alcohol, tobacco, cannabis, and cocaine. The entire VR-CET protocol lasted for 5 weeks, with two assessment sessions (pre-and post-VR-CET and six VR-CET booster sessions). Then, patient A abandoned the treatment received at the Addictive Behaviors Unit. Figure 1 represents a visual overview (VA) of the patient’s relevant current and past clinical data.

Given the complexity of patient A’s psychological status and medical concerns, in addition to the fact that he had an unfavorable response to previous therapies, we aimed at contributing to the educational, scientific, and clinical practice communities by illustrating a novel therapeutic approach and highlighting the clinical potential of technologies like virtual reality and eye-tracking in addressing the underlying mechanisms of AUD.

### Assessment Measures

#### Paper-and-Pencil Instruments (Pre-Post VR-CET Assessment)

*Alcohol misuse patterns* were assessed with the Alcohol Use Disorder Identification Test (AUDIT; Saunders et al., 1993). The Spanish version of AUDIT (Contel Guillamón et al., 1999) is a 10-item scale, and responses to each item were scored from 0 to 4, with a maximum score of 40. A total score equal to or higher than eight points indicates a hazardous drinking pattern and a score higher than 15 is likely to indicate alcohol dependence (Saunders et al., 1993; Higgins-Biddle and Babor, 2018; Nadkarni et al., 2019). Test-retest reliability and internal consistency regarding the original and language-adapted versions of AUDIT present satisfactory properties (de Meneses-Gaya et al., 2009).

*Craving* was explored with the Multidimensional Alcohol Craving Scale (MACS; Guardia Serecigni et al., 2004). The MACS is a Spanish scale assessing the intensity of alcohol craving experienced by the individual throughout the previous week. The scale presents robust validity and reliability properties (Guardia Serecigni et al., 2004). Each item scores ranged from 1 (“Strongly disagree”) to 5 (“Strongly agree”). The MACS has three outcome scores: “desire to drink,” “behavioral disinhibition,” and the total score. Based on responses to the MACS items, these scores were classified as non-existent, mild, moderate, or intense alcohol craving. In addition, an *ad-hoc* modified version of MACS (MACS-VR) was introduced in the protocol to assess alcohol craving after VR exposure. The questionnaire items remained the same as in the original MACS, although the instructions were no longer to assess the “intensity of alcohol craving experienced throughout the previous week,” but to assess the “intensity of alcohol craving experienced during VR exposure.”

*Anxiety* was assessed by the Spanish version of the State-Trait Anxiety Inventory (STAI; Spielberger et al., 1971). The STAI is a self-reported questionnaire with two subscales assessing state anxiety and trait anxiety. Each subscale consisted of 20 items, and scores on each item ranged from 0 (“Not at all”) to 3 (“Very much so”). The Spanish version of STAI has robust validity and good internal consistency properties (Spielberger et al., 1971).

*Attentional bias* was explored with two instruments: the Spanish version of the Alcohol Stroop Test (Sánchez-López et al., 2015) and a VAT. The Alcohol Stroop Test is an adapted version of the classic Stroop task used to assess the interference caused by alcohol stimuli in individuals with problematic alcohol use and AUD. The Alcohol Stroop Test presents good test-retest reliability and good convergent, discriminant and predictive validity (Hallgren and McCrady, 2013).

#### Eye-Movement Activity Data (Pre-Post VR-CET Assessment)

The VAT aimed to explore AB using the ET technology. The VAT stimuli consisted of alcohol-related images vs. neutral images. Alcohol-related images were selected based on the results of the Spanish National Health Survey on Alcohol Consumption (2011/2012). The outcomes of this survey indicated that the most frequently consumed alcoholic beverages were beer, followed by wine and liquors. Neutral images were colorful office-related objects (pencils, sticky notes, writing boards, etc.), as seen in previous research (Castellanos et al., 2009; Donohue et al., 2016). Each image was presented at 960 × 960 pixels. The angle of observation between the patient and the VAT presented on the monitor was 90 cm. The VAT comprised 144 stimuli (12 images related to alcohol × 12 neutral images) and was presented to the patient as a false visual memory task. This false task consisted of 18 trials (eight pairs of images per trial) in which the patient reported whether he had seen a certain image or not in the previously seen trial. All pairs of images were counterbalanced and each pair of images appeared on the screen for 3 s. After each pair of images, a gray background appeared for 1 s with a fixation point in the center of the screen aiming to return to a focal point.

#### Momentary Levels of Craving and Anxiety

Momentary levels of alcohol craving and anxiety were assessed with Visual Analog Scales (VAS). These scales are frequently used as self-reported scales (Bordnick et al., 2008) to indicate craving (VAS-C) and anxiety (VAS-A) during VR exposure. The VAS-C is a self-reported subjective craving scale, with scores ranging from 0 (no craving) to 100 (intense craving). Similarly, the VAS-A is a self-reported scale which explores anxiety levels, with scores ranging from 0 (no anxiety) to 100 (intense anxiety).

#### Urine Analyses

Urine samples were collected tri-weekly during TAU at the Addictive Behaviors Unit from Hospital Clinic of Barcelona, including before, during, and 3 weeks after completing the VR-CET program.

### Instruments

#### Hardware

The *ET equipment* consisted of the Gazepoint GP3 HD eye tracker (150 Hz system, 0.5–1.0 degree of visual angle accuracy, 35 cm (horizontal) × 22 cm (vertical) movement, and ±15-cm range of depth movement) to record eye movement activity, a laptop to register the data, a 19'' monitor to display the VAT at a resolution of 1,280 × 1,024, and a chin-rest to avoid head movements.

The *VR equipment* consisted of an Oculus Rift S head-mounted display (HMD), sensors, touch controllers, and a computer compatible with VR technology (INTEL® Core™ i7–2,600 CPU, 16.0 GB RAM, 64-bit operating system, x64 processor, and a NVIDIA GeForce GTX 1080 Ti graphic card).

#### Software

**Figure 2:**
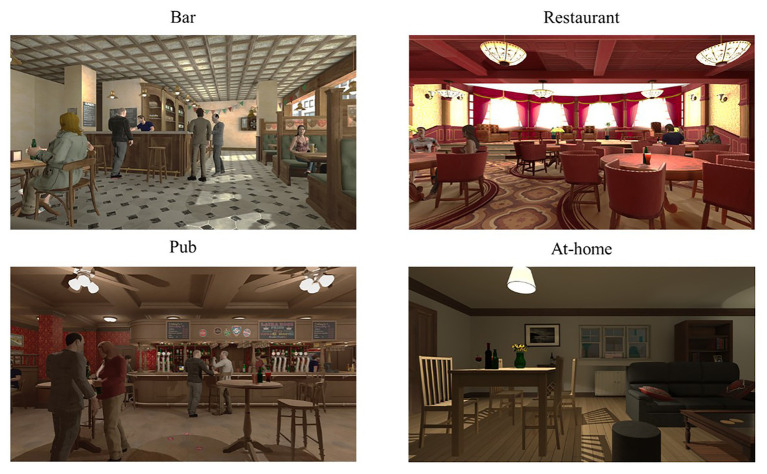
Images of the virtual reality (VR) alcohol-related environments.

The “ALCO-VR” software was created based on the results of a previous study (Ghiţă et al., 2019a) in which we identified alcohol-related triggering stimuli and contexts for craving elicitation in a sample of patients diagnosed with AUD. The software consists of a neutral environment (a room with white background and a glass of water), where users could familiarize themselves with the VR technology, and four alcohol-related environments: restaurant, bar, pub, and at home environments (Figure 2). These scenarios were created considering different variables as in previous research, such as social interactions (including avatars in the environments), different alcohol-related cues (a menu of 22 alcoholic drinks), or different times of day (day time or night time). “ALCO-VR” consists of two parts, assessment and therapy. In the assessment part, users choose their first five preferred alcoholic beverages from the menu of drinks. The software itself was set to create a hierarchy of exposure from the lowest rated environment (context) with the lowest rated alcoholic drink (alcohol-related cue, from the first five preferred drinks) to the highest rated environment and drink. Every 20 s, the user was asked to self-report their alcohol craving and anxiety levels on VASs. The therapy part of “ALCO-VR” was based on the previous assessment part, and involved exposure to significant alcohol-related cues and contexts, as initially selected by the patient. Unlike the assessment part, the therapy system implied self-reports on VASs every 60 s. Only if the ratings were 40% less than the initial ratings on both VAS-C and VAS-A was the user allowed to continue to the next level. The platform permitted a high level of interaction with the VR environments, where individuals approach their alcoholic beverages, grab and observe them from all angles with Oculus Touch controllers.

### Procedure of the VR-CET Program

**Figure 3:**
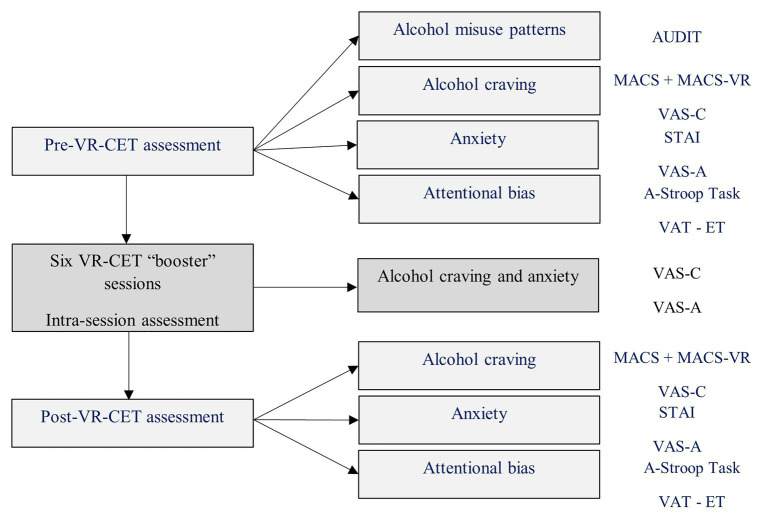
Pre-post Virtual Reality Cue-Exposure Therapy (VR-CET) and intra-session assessment.

Patient A was recruited from the Addictive Behaviors Unit, Hospital Clinic of Barcelona and after providing his written informed consent, he was assigned to the VR-CET group. The initial assessment session consisted of collecting personal data such as AUD history, medication, and abstinence data. The patient was asked to complete the AUDIT (Total score = 28), MACS and STAI (only the trait part), and the Alcohol Stroop Task. Then, AB was assessed with the VAT using the ET technology. Following the VAT, the VR assessment task from the “ALCO-VR” software was administered. A hierarchy based on his preferences was created by the “ALCO-VR” software, and the Oculus Rift HMD was attached to his head. After a short tutorial to familiarize him with the VR technology and the controllers, the patient was asked to rate his momentary levels of anxiety and craving when exposed to the alcoholic drinks (every 20 s) within the VR environments on the VAS-A and VAS-C. After the assessment task, the patient was administered the MACS-VR and the state part of the STAI. Following this initial assessment session, the patient was administered VR-CET sessions twice a week for 3 weeks. Each therapy session lasted approximately 1 h, with 50 min of exposure therapy and generally 10–20 min of psychological debriefing. The sessions consisted of a gradual exposure to the same preferred stimuli and environments as elected in the assessment part, starting with the lowest rated environment and lowest rated alcoholic beverage. During the therapy sessions, the patient was exposed for at least 60 s to each of the preferred alcoholic drinks in each one of the four environments. Hence, every 60 s, the patient self-reported his momentary alcohol craving and anxiety levels on VAS-C (50 ratings) and VAS-A (50 ratings). Three days after completion of the six VR-CET booster sessions, the post-treatment session (the final assessment) consisted of a similar protocol as the initial assessment session. Regardless of the nature of the session (either assessment or therapy), the patient received a short debriefing at the end of each session in order to reduce cravings and to minimize further possible consumption. The assessment and treatment sessions were delivered by an experienced clinician-scientist at VR-Psy Lab, University of Barcelona. With the aim to decrease momentary levels of anxiety and craving and to further minimize the possibility of a relapse, the therapist conducted a brief psychological debriefing at the end of each VR-CET booster sessions (between 10 and maximum 20 min). The debriefing consisted of asking the patient to narrate the situation he was experiencing, including his thoughts, emotions, and behavioral schemas. In addition, each debriefing included deep diaphragmatic breathing for several minutes as suggested by previous research (Priddy et al., 2018). Addressing these concerns, the patient was allowed to leave the laboratory setting only when his responses were classified as “non-pathological” by the therapist. Only when the patient reported momentary levels of alcohol craving and anxiety less than 25 (out of 100), the session was concluded. Figure 3 details pre-post booster sessions and intra-session assessment of the VR-CET program.

### Data Analysis

#### Paper-and-Pencil Instruments (Pre-Post VR-CET)

A similar approach to the ET data was applied to Alcohol Stroop Task and STAI. A positive outcome would be represented by lower scores in attention bias and state and trait anxiety at post-VR-CET assessment session, whereas a negative outcome would be indicated by the opposite patterns (Julian, 2011; Sánchez-López et al., 2015). To determine significant changes regarding alcohol craving as assessed by the MACS and MACS-VR, the total scores were interpreted as: “non-existent” craving [(≤12) which is also the cut-off score], “mild” (13–22), “moderate” (23–40), and “intense” (>40; Guardia Serecigni et al., 2004).

#### The Eye-Movement Activity Data (Pre-Post VR-CET Assessment)

The Open Gaze and Mouse Analyzer (Ogama) software was used to design areas-of interest (AOI) within each VAT image (AOI-alcohol for images with alcohol content, AOI-neutral for images with neutral content), and extract the raw eye-movement data. Within each AOI (AOI-neutral and AOI-alcohol), we computed three dependent variables: dwell time, the number of fixations, and first fixation. These data were transferred to Excel and an additional data transformation was carried out (e.g., computing percentage of each ET variable). McNemar tests were performed for each one of the variables between pre- and post-VR-CET. Regarding the ET data, a positive outcome would mean that the patient displayed the same interest in observing neutral images and alcohol-related images at post-VR-CET assessment. A negative outcome would be indicated by displaying more attention to the alcohol-related images, as well as by displaying more attention to the neutral images (as this last gaze pattern could be the result of an avoidance behavior).

#### Momentary Levels of Craving and Anxiety

Regarding scores of momentary levels of alcohol craving and anxiety, previous research indicated that VASs are a reliable measure to indicate clinically significant changes in cue-induced drug cravings (Ekhtiari et al., 2010; Yeung and Wong, 2019) and anxiety (Williams et al., 2010), with scores between 0 and 25 (“not at all” craving/anxiety), 25–50 (“mild” craving/anxiety), 50–75 (“moderate” craving/anxiety), and >75 (“extreme or intense” craving/anxiety; Reid et al., 1998; Williams et al., 2010; Lundahl and Johanson, 2011). The cut-off score was set at <25 for both VAS-C and VAS-A in our study. At pre- and post-VR-CET assessment, we determined the means and SD of VAS-A and VAS-C in Excel corresponding to the assessment part of the “ALCO-VR” software. Regarding the intra-therapy VASs from the therapy part of the “ALCO-VR” software, we emphasized the highest and final values as clinically significant scores to examine cue-induced craving and anxiety (Pla-Sanjuanelo et al., 2016). In addition, Reliable Change Indexes (RCIs) were computed for MACS, MACS-VR, STAI (state and trait), VAS-A, VAS-C, Alcohol Stroop Task to explore significant changes at post-assessment session following the guidelines of Jacobson and Truax (1991). We also added *z*-scores of the questionnaires: normative data used stemmed from the overall initial clinical sample of 95 subjects from our project (Crawford and Howell, 1998). These scores are detailed in the Results section.

## Outcomes of the VR-CET Approach

### Pre-Post VR-CET Assessments

Table 1 shows the differences in scores as assessed by MACS, MACS-VR, STAI, VAS-A, and VAS-C, and the Alcohol Stroop Task during the first and final assessment sessions. Direct and z-scores from the questionnaires are emphasized in Table 1, alongside the results from the RCIs. Concerning the z-scores, the values obtained show scarce deviations from the mean across the different variables. As for the RCIs, solely the RCIs for both VAS-A and VAS-C variables were statistically significant, therefore expounding that the change was reliable beyond the change expected due to the measurement error alone.

**Table 1:** Pre- and post-treatment scores as assessed by the following instruments.

Measures	Pre-treatment session	*Z* scores (pre-treatment)	Post-treatment session	*Z* scores (post-treatment)	/RCIs/
MACS	31	−0.08	12	−0.56	1.57
MACS-VR	48	+0.31	14	−0.02	1.87
STAI-trait	17	+0.89	7	+0.23	1.87
STAI-state	21	−0.02	4	+0.12	1.92
VAS-A	*M* = 63.3, *SD* = 15.8		*M* = 5.6, *SD* = 4.07		*2.92
VAS-C	*M* = 52.75, *SD* = 17		*M* = 1, *SD* = 1.48		*3.89
Alcohol Stroop Task	70	+0.12	66	+0.19	1.01

Reliable Change Index (RCI) is significant whenever is significant whenever *RCI > 1.96.

### The Eye-Movement Activity Data

Table 2 shows changes in the oculomotor activity patterns at the two moments of assessment, before and after the VR-CET booster sessions regarding dwell time, first gaze, and number of fixations. Despite not reaching statistical significance (Table 3), the data indicated that patient A observed more images with neutral content than images with alcohol-related content at post-VR-CET assessment session as inferred by the three ET variables. The first gaze variable did not follow the same pattern as dwell time and fixations variables. Patient A displayed an implicit, automatic, early-stage attention processing toward the alcohol-related images vs. control images at both pre- and post-VR-CET time points. There was a slight reduction in first fixation toward alcohol-related images at post-VR-CET assessment session. Overall, the patient tended to pay less attention to the alcohol-related images and observing more neutral images at post-VR-CET.

**Table 2:** Eye-movement data recorded at pre-post VR-CET time points.

Eye-tracking data (%)	Pre-treatment session		Post-treatment session	
	Alcohol-related images	Neutral images	Alcohol-related images	Neutral images
Dwell time	46	54	40	60
First gaze	54	46	52	48
Fixations	48	52	42	58

Eye-movement variables: dwell time, first gaze, and number of fixations.

**Table 3:** McNemar tests for the eye-movement activity data.

McNemar test	*p*	Chi-square statistic	OR (95% CI)
Dwell time	0.361	0.833	0.667 (0.293–1.463)
First gaze	0.889	0.019	0.926 (0.515–1.657)
Fixations	0.479	0.500	0.786 (0.428–1.424)

### Momentary Levels of Craving and Anxiety During VR-CET Sessions

**Figure 4:**
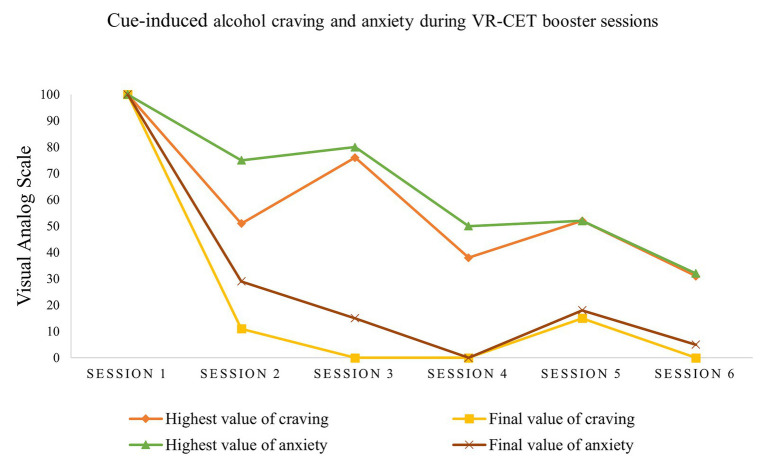
Highest and final craving and anxiety values across the VR-CET sessions.

Figure 4 shows the difference between the highest value and the final value of cue-induced anxiety and alcohol craving in each session and across the six VR-CET sessions. As seen in previous research (Pla-Sanjuanelo et al., 2016), the highest and final values are clinically significant scores to examine momentary levels of craving and anxiety as reported on VAS-C and VAS-A, respectively. During the first session, patient A reported maximum scores of anxiety and alcohol craving on VAS-A and VAS-C (100) at the 10th minute of VR-CET and were maintained throughout the entire therapy session. Following this first VR-CET session, momentary alcohol craving and anxiety levels were gradually reduced. By the end of the therapy sessions, the patient presented lower scores in both highest and final values of craving and anxiety levels. Alcohol craving levels decreased by 100%, whereas anxiety levels were reduced by 95% at the end of the sixth VR-CET session compared to the first therapy session.

### Urine Analyses

In addition, the results of tri-weekly urine analyses were negative and confirmed the patient’s self-reports regarding alcohol and illicit substance use, indicating that he remained abstinent throughout his participation in the study, although tobacco was consumed on an occasional basis.

## Discussion and Conclusion

Our results indicate that VR-CET booster sessions reduced patient’s AUD symptoms, anxiety and craving, and produced a change in gaze patterns. The self-report in the initial assessment session indicated a severe AUD problem according to the AUDIT (Saunders et al., 1993), suggesting that the patient was experiencing a hazardous pattern of alcohol misuse (Donovan et al., 2006). The MACS total score indicated a “moderate alcohol craving” throughout daily life activities, particularly in the previous week before the assessment (Guardia Serecigni et al., 2004). The total MACS-VR score was translated into an “intense craving for alcohol” immediately after the assessment part of the “ALCO-VR” software, as a result of cue-induced alcohol craving (Ghiţă et al., 2019b). These scores were consistent with the self-reported subjective craving on the VAS-C during the VR assessment task. These indicators suggested to us that the patient was experiencing constant alcohol craving, an intense desire to consume, particularly in the presence of alcohol-related stimuli, which may further precipitate a relapse (Pina and Williams, 2016). Interestingly, after the VR-CET booster sessions, patient A self-reported notable reductions on all scores of instruments assessing alcohol craving. Post VR-CET assessment MACS scores indicated “non-existent alcohol craving” in the past week, and the MACS-VR total score reflected “mild alcohol craving” after VR exposure to alcohol-related content. Furthermore, the post-treatment VR assessment task indicated a significant change, almost total absence of alcohol craving as reported on the VAS-C, when the patient was exposed to alcohol-related cues and contexts. In view of these results, we consider that our study depicts the principles of systematic desensitization, in which an individual undergoes a habituation process over repetitive and prolonged exposure to highly salient stimuli (Hernández-Serrano et al., 2020). We highlight the reduction in self-reported subjective craving for alcohol to be important achievements of the VR-CET program. Our findings are consistent with previous research using Positron Emission Tomography/Computerized Tomography, which have shown reductions in craving for alcohol in patients diagnosed with AUD after participation in a 10-session VR therapy protocol (Son et al., 2015).

A similar pattern was found for self-reported anxiety. Although patient A did not indicate a clinically significant score in anxiety (<40), STAI determines the momentary (state) and chronic (trait) anxiety levels and the initial assessment session indicated a higher level of state-anxiety compared to trait-anxiety in patient A (Julian, 2011; Leal et al., 2017). The higher score for state-anxiety was also determined by exposure to alcohol-related VR environments as depicted by previous research (Ghiţă et al., 2019b). These scores were highly consistent with the self-reported subjective anxiety on the VAS-A during the VR assessment task, which was significantly lower when the patient was exposed to alcohol-related cues and contexts. After the VR-CET sessions, patient A obtained notably lower scores on both parts of the STAI, and we emphasize the reduction in state-anxiety (from 21 pre-treatment to four post-treatment). The state-anxiety score fell considerably after the VR-CET sessions compared to the trait-anxiety score, particularly because state-anxiety is a self-regulatory process related to alcohol cue-induced momentary anxiety, whereas trait-anxiety is a more stable personality trait that indicates generalized anxiety levels (Leal et al., 2017).

In terms of AB toward alcohol stimuli, patient A displayed an interference effect on Alcohol Stroop Task for alcohol words, translated as an attention bias for alcohol content. According to the results of Alcohol Stroop Task in the pre-post assessment sessions, we interpret the lower score on the task after the six booster sessions as indicating decreased interference toward alcohol content (Hallgren and McCrady, 2013). Regarding the eye-movement activity, the changes in gaze patterns displayed by patient A after the treatment are interesting. The dwell time and fixation variables indicated a maintenance-stage of attention processing for images with neutral content, translated as a tendency to avoid alcohol-related images at both pre- and post-treatment assessment sessions. Townshend and Duka (2007) explained this avoidance pattern as an increased momentary perception of loss of inhibitory control over alcohol consumption. Patient A showed an increasingly active and conscious avoidance pattern of alcohol-related images and heightened approach eye-movement activity for the neutral content after the VR-CET booster sessions. However, the first fixation variable indicated the opposite gaze pattern. The first gaze variable is an involuntary cognitive mechanism suggesting automatic processing schemas (Schoenmakers et al., 2010). Thus, patient A showed an implicit attention processing translated as an approach tendency toward alcohol-related images vs. neutral images in both pre- and post-treatment sessions. However, after the VR-CET booster sessions, the first gaze variable indicated a slight decreased tendency to regard alcohol-related images and an increased tendency to observe the neutral images. Our findings are consistent with a previous study using the alcohol-visual-dot-probe-task, which showed that after an initial orientation toward alcohol stimuli, AUD patients displayed an avoidance pattern, disengaging from alcohol-related stimuli in the first stages of abstinence (Vollstädt-Klein et al., 2009). This approach-avoidance gaze pattern displayed by patient A has been demonstrated in other studies with individuals with binge eating disorder (Deluchi et al., 2017) or gambling disorder (Ciccarelli et al., 2016).

In terms of intra-session cue-induced momentary craving and anxiety responses, patient A initiated the VR-CET sessions with maximum cue-induced craving and anxiety scores, suggesting a high vulnerability toward alcohol-related cues and contexts, which may increase relapse susceptibility (Fatseas et al., 2018). However, craving and anxiety responses gradually decreased over the course of the six VR-CET sessions, indicating a process of desensitization toward alcohol-related content (Hernández-Serrano et al., 2020). We depicted a slight increase in momentary levels of anxiety and alcohol craving in the fifth VR-CET session compared to the fourth session. Although it was a minor increase, the therapy session coincided with a previous family conflict the patient experienced. According to the patient, this conflict facilitated a “generalized feeling of distress.” Nevertheless, higher and final values of momentary cue-induced anxiety and craving had fallen by the last therapy session, almost reaching extinction of alcohol craving and anxiety responses.

These indicators were consistent with patient A’s daily life experiences, since he reported greater self-efficacy in terms of managing his alcohol cravings and alcohol-related anxiety. This is a fundamental achievement for VR-CET sessions, as the main goal was to generalize this intervention’s effects to the real-life context of the patient. Considering patient’s perspective, he was highly motivated to participate in a study using VR-based therapy. After the intervention, the patient described the sessions as “engaging” and “beneficial” in helping him to cope with real situations associated with alcohol consumption. In addition, the patient highlighted the realism of the VR alcoholic beverages, environments, and avatars as positive aspects of the VR-CET booster sessions. In addition to self-reports using different instruments and technologies, the results of urine analyses confirmed no alcohol or illicit substance use during the VR-CET program and shortly after its completion, only occasional tobacco use. Although the VR-CET program was designed particularly to address AUD-related concerns, we emphasize the clinical potential of the VR-CET program regarding its outcomes in terms of abstinence maintenance.

The outcomes of this case report should be viewed in the light of study limitations. First, the current study reflects a single subject and therefore, solid conclusions about the short- and long-term effectiveness of the VR-CET will be determined by the outcomes of the clinical trial and follow-ups. Second, the patient was under outpatient treatment at the Addictive Behaviors Unit from Hospital Clinic of Barcelona, receiving pharmacotherapy and psychological treatment during his inclusion in the VR-CET program. Nevertheless, we emphasize that patient A underwent several treatments at different hospitals including both psychological and pharmacological treatments, but he experienced relapses after treatment discharges, with maximum of 4 weeks of abstinence each time he attempted to cease substance use. We consider that adding the VR-CET protocol may enhance the baseline treatment received at the Addictive Behaviors Unit and may increase the abstinence period of a resistant to TAU patient. Third, we did not assess other areas of functioning in patient A, as we focused mainly on exploring AB, alcohol craving, and anxiety. For instance, examining patient’s emotional and personality traits would had given a more comprehensive overview of overall patient’s functioning.

Despite promising results, continued research is needed to clearly determine the effectiveness of VR applications in AUD. For instance, randomized clinical trials following the “Consolidated Standards of Reporting Trials” (CONSORT) guidelines are fundamental to establish a rigorous scientific consensus regarding the clinical potential of VR use in AUD. Variables like social interaction implemented in VR environments also raise questions, and it should be addressed to further standardize VR applicability (Ghiţă and Gutiérrez-Maldonado, 2018). In addition, future studies may examine the effectiveness of VR-CET approach alone and in combination with other psychological interventions (e.g., coping skills training, and mindfulness). A recent systematic review highlighted potential limitations of VR use in AUD like cost-effectiveness, training of professionals delivering VR-based programs, or patient’s compliance with the VR program (Segawa et al., 2020). Nevertheless, VR applications in AUD currently follow the promising scientific course of previously validated VR procedures in anxiety disorders (Maples-Keller et al., 2017) or post-traumatic stress disorder (Kothgassner et al., 2019).

## Data Availability Statement

The datasets generated for this study are available on request to the corresponding author.

## Ethics Statement

The studies involving human participants were reviewed and approved by Ethics Committee of the University of Barcelona and Ethics Committee of Hospital Clinic of Barcelona. The patient provided their written informed consent to participate in this study.

## Author Contributions

All authors listed have made a substantial, direct and intellectual contribution to the work, and approved it for publication.

### Conflict of Interest

The authors declare that the research was conducted in the absence of any commercial or financial relationships that could be construed as a potential conflict of interest.
